# Refresh Rate-Based Caching and Prefetching Strategies for Internet of Things Middleware

**DOI:** 10.3390/s23218779

**Published:** 2023-10-27

**Authors:** Alexey Medvedev, Alireza Hassani, Gleb Belov, Shakthi Weerasinghe, Guang-Li Huang, Arkady Zaslavsky, Seng W. Loke, Prem Prakash Jayaraman

**Affiliations:** 1 School of Information Technology, Deakin University, Geelong, VIC 3145, Australia; alexey.medvedev@deakin.edu.au (A.M.); ali.hassani@deakin.edu.au (A.H.); belinda.huang@deakin.edu.au (G.-L.H.); arkady.zaslavsky@deakin.edu.au (A.Z.); seng.loke@deakin.edu.au (S.W.L.); 2 Faculty of Information Technology, Monash University, Clayton, VIC 3168, Australia; gleb.belov@monash.edu; 3 School of Science, Computing and Engineering Technologies, Swinburne University of Technology, Hawthorn, VIC 3122, Australia; pjayaraman@swin.edu.au

**Keywords:** Internet of Things (IoT), middleware platforms, context management, caching, cost efficiency, service level agreement (SLA), optimal strategy

## Abstract

One of the research directions in Internet of Things (IoT) is the field of Context Management Platforms (CMPs) which is a specific type of IoT middleware. CMPs provide horizontal connectivity between vertically oriented IoT silos resulting in a noticeable difference in how IoT data streams are processed. As these context data exchanges can be monetised, there is a need to model and predict the context metrics and operational costs of this exchange to provide relevant and timely context in a large-scale IoT ecosystem. In this paper, we argue that caching all transient context information to satisfy this necessity requires large amounts of computational and network resources, resulting in tremendous operational costs. Using Service Level Agreements (SLAs) between the context providers, CMP, and context consumers, where the level of service imperfection is quantified and linked to the associated costs, we show that it is possible to find efficient caching and prefetching strategies to minimize the context management cost. So, this paper proposes a novel method to find the optimal rate of IoT data prefetching and caching. We show the main context caching strategies and the proposed mathematical models, then discuss how a correctly chosen proactive caching strategy and configurations can help to maximise the profit of CMP operation when multiple SLAs are defined. Our model is accurate up to 0.0016 in Root Mean Square Percentage Error against our simulation results when estimating the profits to the system. We also show our model is valid using the *t*-test value tending to 0 for all the experimental scenarios.

## 1. Introduction

In the current stage of technical progress, the concept of Internet of Things (IoT) has gained significant recognition. However, most of the IoT data are locked in so-called ‘IoT data silos’, which are vertically oriented systems, e.g., proprietary IoT applications. Such systems belong either to an organisation, which uses IoT to control the infrastructure (e.g., road network operators), or to a company that manufactures IoT devices (e.g., fitness bracelets manufacturers as in iWatch, and Fitbit). There is still a lot of hidden capacity to develop services that will use data across several such IoT data silos in a seamless way. The variety of these services cover both real-time applications (e.g., smart vehicles) and offline analytical applications (e.g., smart city planning based on big data). Once created, these services can potentially make our daily lives more efficient and comfortable. To fully realise this vision and enter the IoT ecosystems stage (i.e., the third stage of IoT evolution), IoT requires a horizontal integration of data silos.

A significant amount of research work to enable the development of applications that are dependent on real-time data about external entities has been performed in the area called *Context-Awareness* (CA) [1]. The relevant data, which might also be pre-processed and aggregated, are referred to as *context*. Despite the potential of context-aware computing in multi-disciplinary domain, the progress with introducing these kinds of services in the real world is in its infancy. The main problems are the lack of universal acceptance, standardisation, and accessible technologies [2].

There has been research approaches proposing ways to exchange IoT data in a peer-to-peer way, e.g., [3]. However, these approaches can raise concerns about the scalability, complexity, cost of operation, and safety and privacy control, to name a few. A more feasible approach to the problem can be make data and control flows through a middleware platform, which facilitates interoperability between multiple IoT silos and serves as an aggregator and a redirector at the same time. So, developing a comprehensive and powerful IoT middleware platform is a potential solution.

### 1.1. Motivation for Refresh Rate-Based Caching and Prefetching

*Context Management Platforms* (CMP) [4] are advanced IoT middleware that comprise functionalities such as (i) IoT marketplace, (ii) gateways to multiple data sources, (iii) subscription mechanisms, (iv) features for performing aggregations, reasoning, and analytical functions, and (v) advanced sensor data management. CMPs are designed to provide horizontal connectivity between vertically oriented IoT silos (i.e., proprietary IoT applications). That is, large amounts of IoT data need to be processed in CMPs. Providing relevant and timely services in a large-scale IoT ecosystem requires massive computation and network resources, consequently leading to huge operational costs. The freshness of context (which inversely correlates with the age of context), its accuracy, completeness, and the latency of access to context are several parameters of Quality of Service (QoS). On the other hand, the price that the middleware incurs to access context information is referred to as the Cost of Context (CoC) [5]. Therefore, there is an urgent need to optimize data caching strategies to balance operational costs with the QoS provided. In other words, to maximize QoS and minimize operational costs in a monetised CMP, there needs a model to predict the context metrics and operational costs of context data exchange.

While the IoT, and especially CMP middleware are still evolving [1], the areas of web servers and web proxies have matured decades ago, and the caching techniques used in web servers can already be seen as well-established [6,7]. Moreover, the area of Content Delivery Networks (CDN) and Information-Centric Networks (ICN) is also well-established, and a significant number of techniques can be adopted in CMPs. In particular, the existing caching approaches can be categorized into two main categories: (i) approaches for a fixed size system, e.g., [8,9], and (ii) approaches for systems that can be elastically scaled, e.g., [10]. The first group of approaches is also referred to as *cache replacement strategies*, as the decision is usually about deleting (evicting) an object from the cache when the available resources are close to being exhausted. In the group of approaches designed for fixed-size systems, we can distinguish between *basic caching policies* and *intelligent policies.* However, processing IoT data streams is noticeably different, and balancing the operation cost and QoS requires establishing Service Level Agreements (SLAs) between the context consumer, middleware, and the provider where the level of service imperfection is quantified and linked to the associated costs to find efficient caching and prefetching strategies.

### 1.2. Novel Contributions of This Paper

The main contributions of this paper are two-fold. We develop,

- A mathematical model, which enables predicting the monetary profits of CMP operation in NOD-NOR mode *(short for Not only a Database, Not only a Redirector mode, as we explain later)* [11], when any number of SLAs are defined over a data item, which a CMP provides access to;
- Three main caching strategies for context attributes, which are (i) full coverage, (ii) reactive, and (iii) proactive strategies.

Using the mathematical models, we show how a correctly chosen proactive context caching strategy help maximize the monetary profit from CMP operations when heterogeneous context consumers (emulated using multiple different SLAs) request access to context information.

The paper is structured in the following way. In Section 2, we discuss the efficiency criteria of context caching and management. In Section 3, we provide definitions of the possible caching strategies, which are the full coverage, reactive, and proactive strategies. We show when the proactive strategy can be beneficial; however, this type of strategy is hard to manage due to complexities arising from the stochastic nature of query arrival time. In Section 4, we provide a detailed modelling of processes and develop a mathematical model for proactive caching for cases when one or several SLAs are defined over an attribute. In Section 5, we evaluate the developed methods and show that an adequately chosen refresh period of a cached item can help maximize the profit when several SLAs are defined. In Section 6, we discuss the related work, and Section 7 concludes the paper and sets directions for future investigations.

## 2. Efficiency of Context Storage Management System

One of the hardest challenges faced by the CMPs is processing and managing enormous amounts of context stemming from IoT data providers (e.g., sensors) and other data sources. Self-adaptation is one of the most critical factors for efficient context storage operations. In this section, we discuss three fundamental principles that influence the efficiency of CMP operation, especially in relation to context storage and caching. Then, we formulate the efficiency criteria, which we will use in our modelling.

### 2.1. Modes of CMP Operation and the NOD-NOR Principle

There exist several approaches that to developing a CMP considering how the data flows are managed. We call these approaches (i) the database mode, (ii) the redirector mode, and (iii) the NOD-NOR mode, as described below.

**Database Mode.** The first and, probably simplest, way to organise the data storage and caching in a CMP is to inject all the data from all the connected devices into the storage and respond to incoming context queries using the data from the storage. As the IoT data are transient, the platforms will have to refresh the data at a high rate, causing a high load on the data sources, network, and computation resources. At the same time, serving queries based on the stored data is a more straightforward task, which guarantees lower latencies for the consumer. We call this approach the *database mode*, as all the required data are contained in the internal storage. The difference with the regular database is that the IoT data can potentially be different at the source side when the consumer requests them from the platform. Thus, the main limitation of a CMP working in a database mode is its inability to serve context of the highest freshness. The attempt to achieve maximum freshness inevitably incurs major growth in costs.

**Redirector Mode.** The second way to organise the CMP query processing is to retrieve all the data from external sources on-demand, when the consumer’s request arrives. This approach guarantees the delivery of the freshest context to the consumer. However, it can also cause significant network and computation load. Moreover, retrieving data from external sources causes delays, which results in a long overall latency to serve the context query for the consumer. Furthermore, with this approach, the retrieved data cannot be reused, causing a potential increase in expenses. We call this approach a *redirector mode*, as all the context queries are always redirected by the platform to context providers.

**NOD-NOR Mode**. Trying to tackle the issues possessed by the database and redirector modes, we concluded that the best balance of performance and cost-efficiency could be obtained only by a CMP, that combines the features of both approaches. We called this approach the *NOD-NOR mode (Not only a Database, Not only a Redirector)*.

Most of the CMPs and IoT platforms are designed around a “database” or a “redirector” approach. For instance, FIWARE Orion [12], Nexus [13], and Context Information Service [14] strictly implements the *database mode*. In CoaaS, we have chosen the NOD-NOR strategy, as we believe it is the most cost-efficient way to enable an ecosystem-scale context acquisition. However, our approach is applicable in CMPs operating either in *database* or *redirector* modes because the NOD-NOR strategy is technically a combination of the other two strategies.

For a CMP operating in NOD-NOR mode, the core of self-adaptation is the decision about what data should be kept in the cache for serving queries and what data can be retrieved from external context providers in an ad-hoc manner (when the query arrives). Biasness to caching or retrieving in an ad-hoc manner is a function of quality requirements, features of the context query load, features of the context providers, features of the data streams, and context information [15]. Consequently, we can state that efficient proactive context cache management is one of the impending challenges for any context storage including CSMS. The effective realisation of the NOD-NOR mode in a CMP is a challenging task, requiring significant research efforts. First, software development requires significantly more effort due to the increased complexity of the data flow. Secondly, the NOD-NOR approach requires efficient self-adaptation mechanisms, which are based on predictive models.

### 2.2. Deployment in Distributed Environments

The second fundamental pillar of the current research is the deployment of Context Management Platforms (CMP) in distributed environments. In the past, the designs of adaptive algorithms were aimed at optimising the performance of fixed-size systems or finding the right size for the needed system. Examples of this approach are LRU, LFU [16], and other methods described in Section 6. However, with the rise in IaaS and PaaS business models [17] in the Cloud, and upcoming edge and fog computing paradigms [18,19], it became possible to quickly scale the system to suit the changing requirements. For instance, in a manually configured Cloud deployment, a systems administrator can promptly allocate several more Cloud instances for a NoSQL database and reconfigure the load balancer to use these additional instances as shards. By this, an administrator can achieve lower query execution times, or a greater number of parallel queries served per second. The described approach is called horizontal scalability.

In a Cloud system, for instance, achieving higher performance will come at the cost of paying for these additional resources (e.g., servers, storage). However, when the need to serve an additional amount of queries disappears, an administrator must deallocate some of the Cloud instances to reduce the operational cost. If the pattern of incoming load (the number of queries at a particular time of the day) is known, the process of allocation and deallocation can be automated.

Obviously, not all computational tasks can be easily scaled. For instance, the operation of RDBMS is well known for issues with horizontal scaling, i.e., post-scaling performance degradation [20]. We have designed the modules in Context-as-a-Service (CoaaS) in a way that is compatible with the concept of horizontal scalability to enable the CMP to operate in distributed environments and be adaptable to varying context query loads. Adapting the approach in this way brings us to an understanding that the cost of caching (the use of distributed resources and external services), together with meeting the necessary constraints will be crucial in deciding the context caching strategy.

### 2.3. Transient Nature of IoT Data

Now we can move to the third pillar of current research, which is the *transient nature of IoT data.*

IoT data consist of a large number of relatively small-in-size data, which we will also interchangeably call *context attributes*. These raw attributes are used to deriver higher-level context using aggregation and inferencing techniques on the raw IoT data, e.g., the smallest possible datum is a measurement with a timestamp from a remote sensor.

The main difference between IoT data and other types of data is that IoT data change over time, or, at least, they can change. For instance, a car can change its location with time when the vehicle is used, or the location will remain the same while the vehicle is parked. In this case, the location is an example of a continuously changing attribute. An attribute can also be binary; for instance, the occupancy of a particular parking spot. An attribute can also take a value from a defined set (e.g., red, yellow, green). The critical point is that IoT data can become obsolete *at any time*. A newspaper article or a video file, on the contrary, are examples of non-IoT data typically dealt with in caching systems. Such data will not usually change over time. If we look at IoT data from another perspective, very strictly, we can say that any IoT data that are returned to a consumer can become obsolete, as they take at least several milliseconds to transfer from a provider to a consumer. During these several milliseconds, a real value (in a sensor) could already be changed. In the best (and the most expensive) case, the speed of data transfer is limited by the speed of light.

A counterargument could be to use techniques where the context provider push data asynchronously when there is a change in reading (i.e., report-by-exception). Apart from such techniques supporting only the database mode (discussed in Section 3), they still do not alleviate the age of context during transmission. However, we cannot ignore the fact that the provider set freshness is most reliable until the provider is adequately dependable—based on the consumer’s context requirements (e.g., sensors can be unreliable due to wear, or dust/residue accumulation on receptors). Hence, quality-based selection of providers at retrieval time [5] and techniques such as alternate context provider retrievals [15] can be required to maintain the cost and quality of context. The role of context providers in determining the Quality of Context is out of the scope of this paper because, (i) CMPs such as CoaaS rely on cost and quality-based context provider selection mechanisms prior to retrieving IoT data [5], and (ii) assuming the providers and state-of-the-art protocols such as CoAP and AMQP are fast and reliable.

However, a consumer is not interested in obtaining a value that absolutely matches reality, as, strictly speaking, absolute matching is impossible in general. Usually, a consumer is satisfied with some level of confidence. As we move from real-time critical systems towards smart city cross-domain scenarios, which inspire the development of CoaaS and other CMPs, we can see that the level of needed confidence is reduced. For example, it is enough to know that a car is moving in a specific area and direction, without knowing the exact place, or it is enough to know that in a certain parking area, there are about a hundred available parking spots, and there is no need to know when a value changes from 100 to 99 vacancies or a specific spot is occupied. However, a data item will lose its freshness with respect to time yet will remain useful for many or some use cases. Every use case must define its need individually, therefore.

Negotiating the level of freshness and other Quality of Service (QoS) and Quality of Context (QoC) parameters between context consumers and a CMP is usually carried out by defining the Service Level Agreements (SLAs). While commercial Cloud IoT platforms have defined the usage of resources in their own terms for their purposes, the definition of SLAs for CMPs should be based on different principles and is still in its infancy. Some efforts have already been made [21]. Nevertheless, we can create a minimalistic SLA by defining just four key parameters: (i) maximum acceptable time to serve a context request, (ii) minimum acceptable degree of freshness (i.e., minimum confidence about a piece of context information after sampling and/or inferencing) (iii) price of a context response, and (iv) penalty paid in case when a request is not served within a defined time. We provide a more detailed description of each SLA parameter further in this section.

To sum up the discussion above and apply it to CoaaS’s cache management framework, we need to fuse the objectives pursued by a CMP with the three main principles of CSMS operation: (i) NOR-NOD mode, (ii) non-fixed size of a system, and (iii) SLA definition based on freshness, where multiple levels of SLAs are possible.

The main objective is to minimise the cost (or to maximise the profit) of a CMP operation. In the long-term, the cost of serving incoming Context Definition and Query Language (CDQL) queries must be lower than the revenue acquired from the consumers. All other objectives are subordinate to the main objective and are reflected in the SLAs with consumers. These secondary objectives are: (i) reducing the time of serving queries and (ii) keeping the acceptable level of the QoC, which is, foremost, dependent on freshness.

### 2.4. Definition of Context Storage and Caching Efficiency

First, we need to define the concept of efficiency in Context Storages (which includes context caches). As a platform, e.g., CoaaS, connects two types of entities: (i) *context consumers*, and (ii) *context providers*. Similar to the real world, consumers want the best possible service delivered free of charge and without any latency. Providers have constraints on their physical abilities (latency, throughput) and, potentially, want to receive payments for providing the sensing services. In the middle of this IoT ecosystem, CoaaS or any other IT solution is operated by a private or public company. Consequently, the platform operation cost (losses) must be balanced with the received income, so that the overall service profit would be reasonable and predictable.

For a single context query, the income is easily defined as a price paid by the consumer for having a query serviced correctly, i.e., meeting the QoC, and QoS requirements, cost expectations, and in a specified time. The loss is defined as the sum of several costs: (i) the price of remote services being called, (ii) the amount of cloud service resources (processing, storage, network) being used by the platform, (iii) the amount of penalty which is returned to the account of the consumer when a query is not serviced in a specified time. Examples can be found in [15,22]. Moreover, the administration of the platform can add any other constraints (e.g., maximum percentage of failed queries for a particular consumer). Eventually, according to the discussion above, we have defined *efficiency as relating to the optimal point of the platform’s operation in terms of operational costs under certain circumstances (i.e., the defined SLAs, and estimated provider behaviour).*

Defining efficiency in such a way allows us to proceed to the next step in the modelling process. The basic form of context is a simple data item (also referred to as context attribute). A data item is an atomic value, for example, a sensor reading. The most important characteristics of the data item which is used to serve a query are (i) the latency, required to access the data item, and (ii) the correctness of this data item. In general, according to the NOR-NOD concept, it does not matter to a consumer where the data come from when responding to a context query—e.g., from a remote source or the context cache, as long as they are dependable and relevant to the consumer. Consequently, all the storage of transient (non-static) IoT data can be viewed as a cache.

We should also take into account the role of a CMP (facilitating horizontal integrations), and the difference from real-time critical systems, which are designed for time and mission-critical applications. In a CMP, a certain amount of inaccuracy and latency can be allowed if it is balanced by reasonable quality and cost as we discussed in Section 2.3.

Consequently, we have adopted the notion of “freshness” [23,24] which reflects how far a data item could deviate from the real value. The freshness can be estimated by monitoring the behaviour of a data item for a while [25]. There is a vast body of knowledge applicable to predicting the behaviour of a value, based on time series analysis. However, these methods are uniquely defined for different types of data (i.e., binary, continuous) and will not be considered in this paper.

We assume that the expiration time is assigned to a data item so that that freshness will decrease from 100% to 0% (or from 1.0 to 0.0). It is always known to the platform in which state of freshness the data item is in. Loss of confidence in a piece of context is non-linear based on [26] where $g\left(∆\right)$ is the age penalty function of ∆—context age. $g\left(∆\right)={∆}^{a}$ or $g\left(∆\right)=e^{a∆}$ where *a* is a scalar. However, for simplicity, let us consider the freshness decay linearly. In other words, it is always known in advance what percentage of freshness the data item will have at any point in time. Hence, to define an SLA between a consumer and the platform, we just need to negotiate four main points: (i) the degree of acceptable freshness, (ii) the price of serving a request, (iii) the acceptable delay, and (iv) the penalty in case the query is not served in a specified amount of time.

As the CMP is operating in a distributed environment, where the number of resources is effectively unlimited, we can potentially cache all the related data and refresh every data item at a very high rate. However, running a system with such a strategy will require an extremely large set of resources and then afford to pay for the infrastructure and the calls for remote services. Caching can undoubtedly help to reduce the number of expensive calls to remote providers as well as to reduce the query serving time. The disadvantage of caching is the loss of freshness and the increased cost of storage and processing resources, as well as the complexity added to the system. Thus, the decision to be made is which items stored in the cache *Need To be Refreshed* (*NTR*).

*NTR* is a scalar value (∈ℝ) that denotes the necessity to refresh a context immediately. That is, higher the *NTR* value, higher the context is required to be refreshed in order to maximize the quality of context (i.e., freshness). *NTR* being a value-based approach, we need to define a threshold above which an immediate triggered retrieval is performed and refreshes the context. The parameters involving the *NTR* and the formula to calculate are discussed below.

### 2.5. Parameters Influencing the Cache Decision

**Figure 1:**
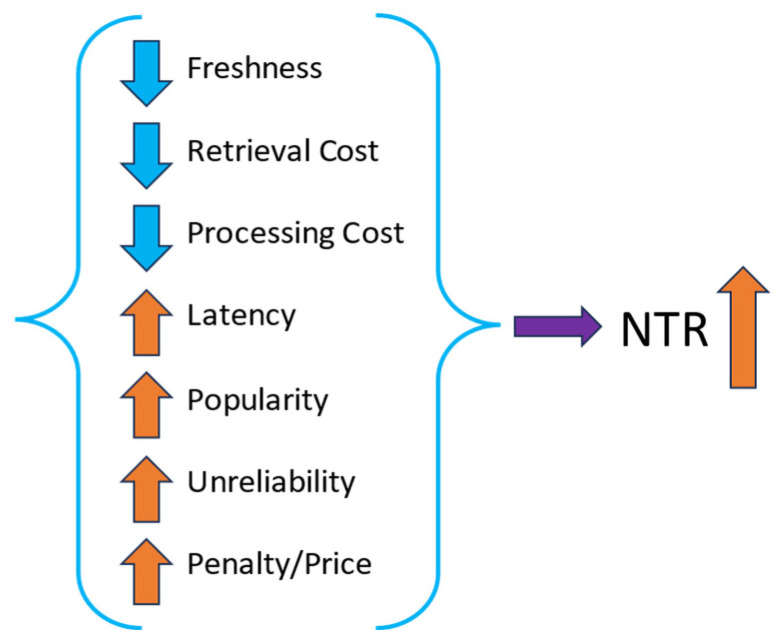
Parameters influencing the NTR and how they impact on increasing the NTR.

The main criteria initially used for the NTR strategy consist of the following six items: (i) Freshness, (ii) Latency, (iii) Popularity, (iv) Retrieval cost, (v) Unreliability (Possible unavailability of a context provider), and (vi) Processing cost. Graphically, their influences to increase the NTR are presented in Figure 1. The up arrows indicate the parameters, which are directly proportional to the value of NTR. The down arrows indicate the parameters that are inversely proportional to the value of NTR. In particular, when the freshness, the retrieval cost, and the processing cost are decreasing, the NTR grows. On the contrary, the NTR grows when the latency, popularity, unreliability, and penalty-to-price ratio increase.

**Freshness** can take a value between 0 and 1. It can be estimated as a result of monitoring the historical values of a particular data item, i.e., the context lifetime [25]. Freshness close to 1 shows that a data item is considered to be very reliable (just fetched from a provider).

**Retrieval cost** is the amount of money that is charged by the data provider for using its API. For instance, the Google Weather API provides information for free only for a relatively small number of calls per day. For a production-scale system, the number of calls will be significantly higher, and each API call will have a cost.

By **processing cost**, we mean the cost of networking, storage, and computational resources that are consumed by a CMP to perform all the needed computations over a single data item when it is retrieved.

**Latency**. Any communication with a remote data source introduces unavoidable latency. However, the value of such latencies can significantly vary depending on the (i) distance to the provider, (ii) the quality of the network and its load, (iii) the number of redirects and routers, and (iv) the number of requests that a provider is serving at a particular time, and many other technical issues. In general, high latency significantly reduces the possibility of obtaining data from a provider on the fly while serving an incoming query. It means that a particular data item, which is retrieved through a slow connection, is to be kept and maintained in the cache.

**Popularity**. From the business perspective, CMPs aim to keep customers satisfied by providing a reasonably high quality of service for a reasonably low cost. The first question to answer is how popularity affects the overall NTR value of a data item. If an item is popular (and is predicted to be popular), it means that the item will be used to serve many context queries and, in turn, definitely requires caching. Otherwise, the number of external calls will be very high causing latencies, network overloading, and high cost of retrieval and processing.

**Unreliability.** As we are dealing with IoT entities serving as context providers, we should assume that some of these entities (e.g., mobile) can be connected to the network via unstable and slow (wireless) channels. All these factors lead to a high possibility of a context provider becoming unavailable during a certain time-period due to network bottlenecks or handover issues. The unavailability of a data provider, in the case of having no valid data in the cache, will cause CoaaS to either exclude the data from a particular provider from the result set or increase the overall time of serving the query while trying to establish a connection or at least select and retrieve from an alternative context provider in the end (which requires invalidating existing cached context and re-inferencing using the new data where necessary [15]). All these consequences are not acceptable especially if high QoS is needed. Therefore, we suggest that low reliability (high probability of node unavailability) will be a reason for the higher caching rate (cache more often).

**Penalty/Price ratio.** The price of a data item access is the price that a context consumer pays to retrieve a data item. Penalty, on the other hand, is the amount of money that is returned to the consumer account when a data item request is not served in a specified period of time. The price of access to a data item and the penalty are defined in the SLA. The higher the penalty (while the price is fixed), the higher the penalty/price ratio, and, consequently, the greater the need to refresh a data item, as not being able to properly serve a query will cause substantial losses.

While the NTR definition helps illustrate the concept, it is not very practical. NTR decisions can become obsolete very fast. In practice, it is better to compute the optimal refresh rate for all the data items, and then periodically check if the rate must be adjusted. Further, defining a unified threshold for all context is unsuitable since the heterogeneous context has different refreshing requirements. Yet, defining unique thresholds for each context and adapting them with time involves a massive monitoring and evaluation process that is computationally expensive as there is theoretically no limit to the different context that consumers may try to access.

In the next section, we analyse the refresh rate-based approach and corresponding strategies in detail. Then, we propose the methodology for cost prediction of a planning period for the refresh rate-based approach, taking the possibility of multiple SLAs into account.

## 3. Refresh Rate-Based Caching Strategies

In this section, we describe three refresh-rate-based caching refreshing strategies, useful for managing cached context attributes. These strategies are (i) the full coverage strategy, (ii) the reactive strategy, and (iii) the proactive strategy. The strategies correspond to the database mode, redirector mode, and NOD-NOR (Not Only Database, Not Only Redirector) modes of CMP operation. Each of the strategies can be beneficial in certain circumstances, as we show below.

We use the following notation in diagrams throughout the paper: a vertical orange arrow represents a retrieval of fresh data by CoaaS from a context provider; a vertical dark blue arrow represents a request to a data item arriving from context consumers to CoaaS at random moments. We assume that the arrivals of requests are happening in accordance with the Poisson distribution. Horizontal dotted lines represent the SLA levels of freshness of a data item, which is stored in CoaaS. The dotted line which is shown at the level of ‘*Freshness = 1′* represents the level of maximum freshness when the data item is just retrieved from a provider and cached in CSMS. The diagonal dark green lines, which are going diagonally downwards from the top of the orange arrows, represent the loss of freshness of a data item over time (which we assume is linear). The second horizontal dotted line represents the SLA threshold of freshness. A data item with a freshness level below this threshold cannot be reused when a request arrives from a context consumer. The height of the dark blue arrows (requests) depicts the desired level of freshness. The time between the level of maximum freshness (item just retrieved) until the moment of reaching the SLA threshold is called the expiry period (or freshness period), which is designated as *ExpPeriod* in the diagrams and as *T* in subsequent mathematical expressions. In the situation when several SLAs are defined, expiry periods differ for each SLA.

The time between retrievals is called a *refresh period* and is designated as *Refr. Period*. We distinguish the *planned refresh period* from the *real refresh period.* A *planned refresh period* is the time between planned retrievals of a data item by CoaaS from a context provider. A *real refresh period* is the average time between retrievals. The real refresh period can be smaller than a planned period, as possible cache misses can trigger the immediate refreshing of a data item.

Another important notion is the *planning period*, which is designated as PlanningPeriod in subsequent mathematical expressions. The *planning period* is the period of time during which the decision about the caching strategy and the corresponding parameters are made. The planning period can contain many refresh periods.

### 3.1. Full Coverage Strategy

**Figure 2:**
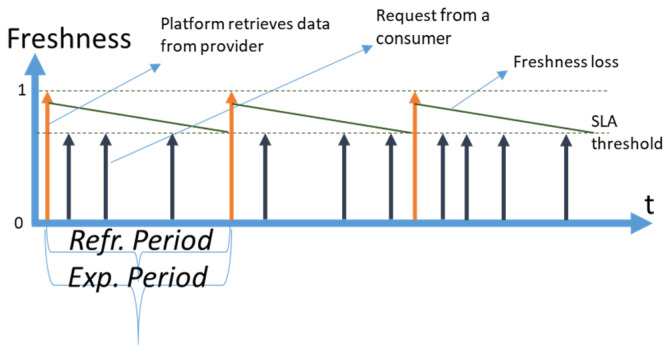
Full coverage context refreshing strategy.

The first strategy is full coverage, which is graphically represented in Figure 2.

With a full coverage strategy, CoaaS is always able to serve a request to a particular context attribute out of the cache memory. In some sense, it can be viewed as a database mode of operation, where freshness is taken into account, e.g., [25]. The difference full coverage has with the database mode is that all the data contain the latest sensor readings in a real IoT database, while in a CMP’s cache, the attribute value contains a sensor reading that is fresh enough to cover the most an SLA with the shortest expiry period (most expensive SLA). When the freshness of a context attribute becomes too low to serve a request for any SLA, a new attribute value is retrieved from the context provider. Consequently, the *maximum reasonable refresh rate* (MaxRate) is the rate at which CSMS refreshes a data item as soon as its expiry period for the SLA with the shortest expiry period elapse:

(1) $$MaxRate=\frac{1}{ExpPeriod}$$

Refreshing at a higher rate will not improve cost-efficiency. On the contrary, it will reduce the efficiency, as the cost of retrieval will increase. With full coverage strategy, the penalty component will → 0, as it is always possible to serve a request out of the cache. The estimation of the profit of operation for CoaaS during a planning period is trivial:

(2) $$\begin{array}{ll} Profit\left(FullCoverage\right) & \\ & =\lambda \times PlanningPeriod\times PriceReq\\ & -\left(\frac{PlanningPeriod}{ExpPeriod}\right)\times PriceRetrieval \end{array}$$

In the expression above, λ is the arrival rate of requests in Poisson distribution, which represents the popularity parameter. The arrival rate and the length of the planning period should be measured in the same unit. For instance, in our simulations, we use the number of requests per second to characterise λ and the planning period is in seconds. PriceReq is the price that a consumer pays for each request and PriceRetrieval is the price which CoaaS pays to retrieve a data item from a context provider. Note that $\frac{PlanningPeriod}{ExpPeriod}$ is the number of retrievals within the *PlanningPeriod*. From here onwards, we assume that PriceRetrieval includes both the price of the accessing the API of the external provider and the cost of internal cloud resources used to process the data item. The total profit of operation for the full coverage strategy (Equation (2)) consists of one positive component and one negative component. The positive part is the income received by CoaaS while serving a certain number of expected requests for a specific price. The negative component is the number of retrievals multiplied by the cost of retrievals.

### 3.2. Reactive Strategy

**Figure 3:**
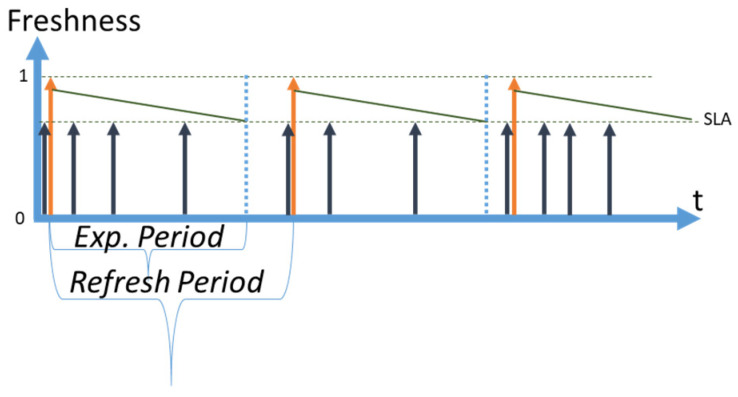
Reactive context refreshing strategy.

The second strategy is the reactive strategy, which is graphically represented in Figure 3. The reactive strategy is conceptually the opposite of the full coverage strategy. The refresh rate is set to zero, meaning that CSMS does not retrieve data proactively. A retrieval happens on the fly only in the case of a request arrival, that causes a partial cache miss [22] (which we will generalize as *misses* in this paper). In this case, the moment of planned retrieval is set to infinity (i.e., never).

In Figure 3, a request from a context consumer to CoaaS (dark blue arrow) happens before the retrieval from an external provider (orange arrow). During the expiry period, all the arriving requests are served out of the cache. When the freshness (green diagonal line) reaches the SLA threshold, (this moment is shown with a dotted vertical blue line), the cache is not covering the data item anymore. When a new request arrives after the moment of expiry, (blue vertical dotted line), it causes immediate retrieval (the orange arrow occurs immediately after the request). Then, the process starts again. Every request that causes a retrieval is a cache miss, which incurs a penalty (refer to Section 2.5). At the same time, every request that arrives during the time when the cached data are fresh (i.e., still above the SLA threshold), is a cache hit. The percentage of hits is called the hit rate, and the percentage of misses is called the miss rate. We designate the hit rate as HR and the miss rate as MR. As can be seen from the figure, the expiry period and the refresh period are not equal, different from the full coverage strategy. The refresh period depends entirely on the arrival of the first cache miss, making it random. As such, we use the average refresh period over a planning period.

We can estimate the cost of the planning period for the reactive strategy as follows:

(3) $$\begin{array}{ll} TotalCost\left(Reactive\right) & \\ & =\lambda \times PlanningPeriod\times PriceReq\\ & -\lambda \times PlanningPeriod\times MR\left(T\right)\times PriceRetrieval\\ & -\lambda \times PlanningPeriod\times MR\left(T\right)\times Penalty \end{array}$$

The expression above (Equation (3)) differs from the expression for the full coverage strategy (Equation (2)) as the second component represents the price paid by CoaaS for retrievals as a result of context cache misses represented by the miss rate (MR). The third component represents the loss caused by penalties, which is also expressed using the miss rate.

Hit and miss rates for a reactive strategy depend only on the arrival rate of requests (λ) and the expiry period of a data item (*T*). The hit can be computed as described by Jung et al. [27]:

(4) $$HR\left(T\right)=\frac{E\left[N\left(T\right)\right]}{E\left[N\left(T\right)\right]+1}$$

In the expression above, *E*[*N*(*T*)] is the expectation of requests that arrive during the expiry period of a data item *T*: $E\left[N\left(T\right)\right]=\lambda \times T$. Accordingly, based on the fact that *MR*(*T*) = 1 − *HR*(*T*), we can also compute the miss rate as follows [27]:

(5) $$MR\left(T\right)=\frac{1}{E\left[N\left(T\right)\right]+1}$$

The hit rate (HR) and miss rate (MR) are always less or equal to one. The total number of retrievals during the planning period (RetrievalNum) depends on the miss rate and the expected number of requests (RequestNumber) and can be computed as:

(6) $$RetrievalNum=MR\left(T\right)\times RequestNumber$$

### 3.3. Proactive Strategy

**Figure 4:**
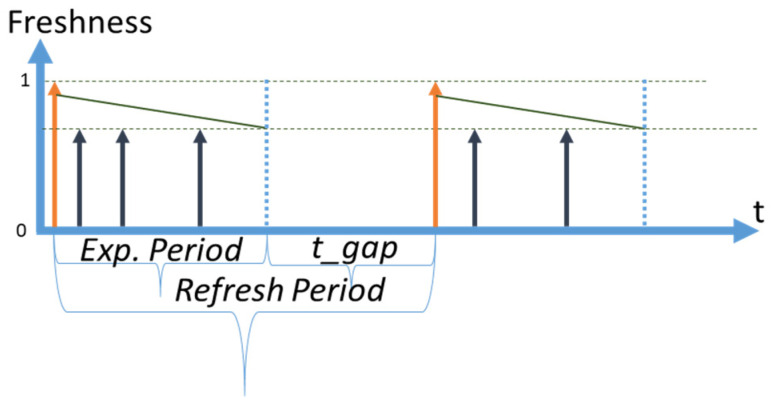
Proactive context refreshing strategy.

The third strategy is the proactive refresh strategy. With this strategy, the moment of planned retrieval happens after the expiry period of an SLA with the shortest expiry period (the most expensive SLA in this paper), but before t = ∞. We call the time Δt between the end of the expiry period and the moment of planned retrieval a “***gap***”. If a request arrives during this period, it “falls within the gap” and CSMS has a cache miss. The proactive strategy is the most sophisticated approach, as it requires adaptive mechanisms to estimate the optimal size of the gap to achieve the most efficient operational cost. We designate the size or the time of the gap as *t*_*gap*. A diagram illustrating the proactive strategy and the concept of the gap is presented in Figure 4. As can be seen in the figure, CoaaS proactively retrieves a data item and requests are served out of the cache till the moment of expiry. The time elapsed from the moment of expiry (blue dotted vertical line) to the moment of the next planned retrieval (orange arrow) is the gap and the size of this gap is shown as *t_gap.*

Figure 4 depicts the fortunate scenario where no requests are arriving during the gap. In this case, the *refresh period* is the same as the *planned refresh period*, which is equal to the sum of the expiry period and the gap size.

However, if there is a request arrival during the gap, a cache miss will occur. There are a number of options to handle cache misses during gaps including: (i) immediate retrieval to serve the request and no reuse of the retrieved data item (no reuse), (ii) immediate retrieval and reuse until the next planned retrieval (reuse without shift), and (iii) retrieve and shift the time of the next planned retrieval (reuse with shift). Technically, it is possible to define more options, such as no retrieval and waiting until the moment of the next planned retrieval.

**Figure 5:**
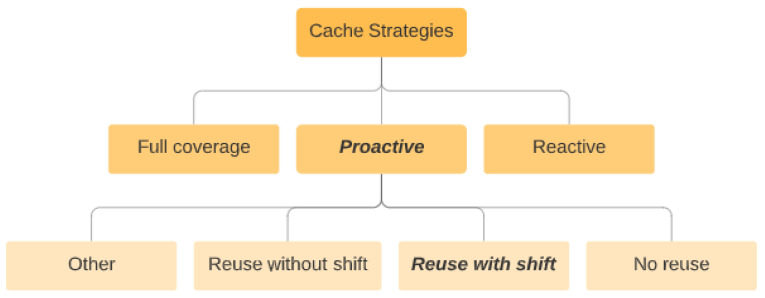
Classification of different context cache refreshing approaches.

**Figure 6:**
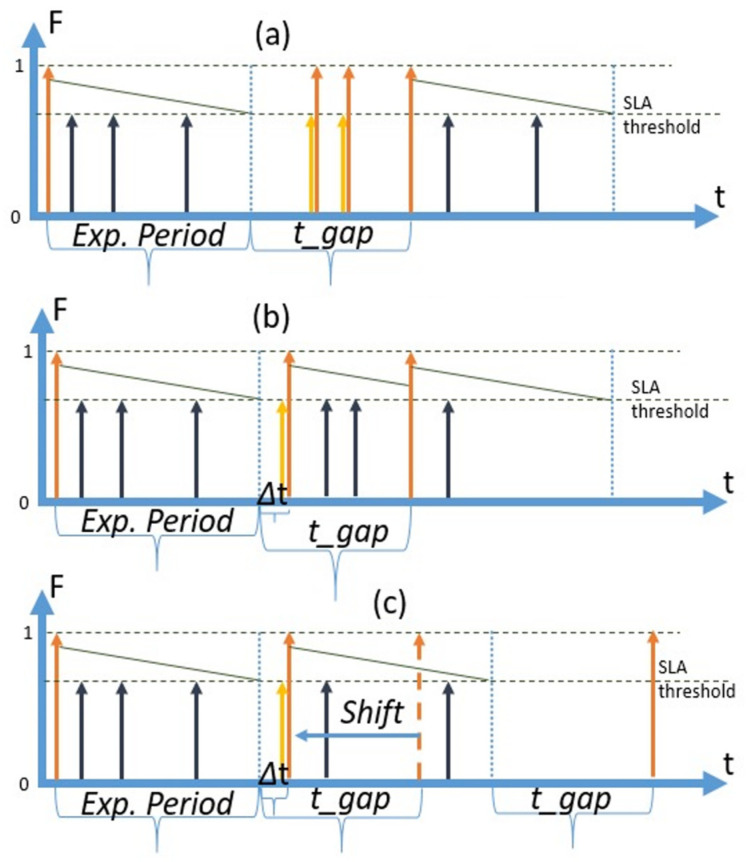
Approaches to dealing with triggered retrieval-induced cached context when proactively refreshing: (**a**) no reuse, (**b**) reuse without shifting, and (**c**) reuse with shift.

The classification of possible options to deal with retrievals during the gap is presented in Figure 5. We have designated the strategies, which were in the focus of the current study with the bold font. The diagrams of these options are presented in Figure 6.

The “no reuse” option is depicted in Figure 6a. Requests which arrive during the gap (shown with yellow arrows) cause retrievals. However, the retrieved items are not cached (not reused) and each new request causes yet another retrieval. At the end of the gap, a proactive retrieval is then initiated by CoaaS.

The “reuse without shift” option is shown in Figure 6b. In this case, if a request arrives during the gap, it causes a retrieval, and the data item is cached. When the planned gap expires, CoaaS proactively retrieves a data item even though the cached item is still fresh enough to serve queries.

The “reuse with shift” option is shown in Figure 6c. The difference between this and the previous option is that after the cache miss and data retrieval the planned retrieval time is changed (shifted). The initial planned time is shown as a dotted orange arrow. As the planned retrieval is shifted, the initially planned retrieval is cancelled, and the gap time starts from the end of the expiry period.

While options (a) and (b) have some benefits from the perspectives of technical simplicity resulting in more straightforward planning. The ultimate cost-efficiency, however, can be achieved with the option (c), as only this option allows reuse during the entire freshness period. Consequently, we have chosen option (c) for our studies of proactive context cache management. In subsequent discussions, the term “proactive strategy” will mean a “proactive strategy option reuse with shift”.

**Figure 7:**
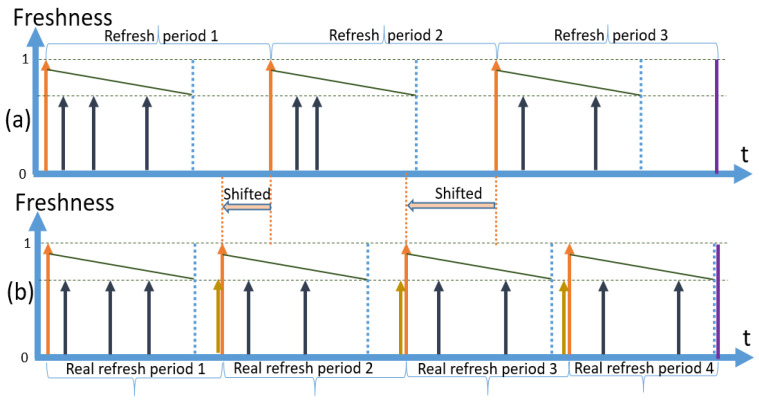
Planned and real retrievals in the long run (**a**) where no queries arrive during the gaps, and (**b**) where queries arrive during the gaps.

To elaborate further on option (c), the concept of *shifted retrievals* happening during a planning period is graphically represented in Figure 7. In Figure 7a, the planning period is depicted, where no query arrivals happen during the gaps. The end of the planning period is represented by a vertical purple line. As everything goes according to a plan, there are three complete refresh periods fitting into one planning period. In the second diagram (Figure 7b), a more realistic situation is shown.

Requests arrive during the gap, causing retrievals to happen before the planned moment. We call this a *shift*. Eventually, in the current example, four periods fit into one planning period, as the real refresh periods are shorter than the planned refresh periods.

### 3.4. Strategy Considerations with Respect to Multiple SLAs

For a situation with a single SLA (i.e., 1SLA), the optimal (most profitable for CoaaS) strategy is either full coverage or reactive. This means that using the proactive strategy and planning the gap is always suboptimal. However, there are two fundamental issues.

Firstly, we still need to decide between the reactive and the full coverage strategy which requires estimating the cost of operation in both cases. While it is clear for the full coverage (Equation (2)), it is not as evident for the reactive strategy. The main issue with the reactive strategy is (as at the moment) when there is a cache miss (and a triggered retrieval), the process of miss and retrieval starts again. Eventually, it is not obvious how to compute the cost of operation for the planning period since there will be hits, misses, and retrievals involved. We have demonstrated this in Equation (3).

**Figure 8:**
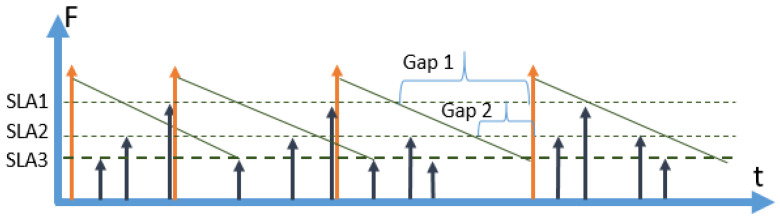
Gaps result in one or more consumers not meeting the freshness requirement specified in the SLA (multiple SLA scenario).

Secondly, there is always a gap time between the freshness requirements of the SLAs unless the freshness requirement of the most expensive SLA is chosen as a strategy among more than one SLA applicable to the context. This concept of gaps that appears in the case of multiple SLAs is graphically presented in Figure 8. In this example, we have decided to provide full coverage only for SLA3 (designated with bold dotted line), which has the longest expiry period, compared to SLA1 and SLA2. In the third refresh cycle, there are no cache misses, and the full gaps for SLA1 and SLA2 are marked. We have provided this example to show how the gaps will inevitably appear in the case of a policy with multiple SLAs unless full coverage is provided for the shortest expiry (or most expensive) SLA. In a policy with multiple SLAs, the shift will happen in the same way as in a policy with a single SLA. When there is a cache miss and a data item is retrieved on the fly, the whole process shifts to the left on the time axis, making the real number of refresh periods higher than the number of planned refresh periods as we presented in Figure 7b. Consequently, there is a need to estimate the cost of a planning period considering the number of hits, misses, and refreshes.

In this section, we have described three main strategies of caching in CSMS. For the proactive strategy, we have also described the concept of gaps as well as our approach to handling cache misses when they happen during the gap. We have illustrated our concepts on a policy with one SLA, as it is the most basic level. As we have shown, the decision process for a single SLA policy is not excessively complex in practice. However, there is still a need to develop a method to estimate cost-efficiency in a situation with gaps. Moreover, in the situation where multiple SLAs are defined, deciding the optimal caching rate becomes much harder. So, a method to predict cost becomes even more important.

In the next section, we will discuss and present our approach to predicting the planning period cost for policies with single SLA and multiple SLAs.

## 4. Predicting the Profit for a Planning Period

In this section, we address one of the most important questions—the cost prediction of a planning period when a proactive strategy is chosen. At first, we formally define the main notions and convey a generalised formula for cost estimation, when multiple SLAs are defined.

Let *SLA* = {*SLA1*, *SLA2*, *SLA3*…*SLAn*} be the set of defined SLAs; each member of the set contains cost-related components, defined in Section 2.5. These components are (i) the request price, (ii) the retrieval cost (which consists of the service call cost, and the processing cost), and (iii) the penalty cost.

We can formulate a generalised formula for predicting the profit of a planning period:

(7) $$\begin{array}{ll} ProfitOfPlanningPeriod\\ & =\underset{s\;\in \;SLA}{\sum }RequestIncome_{s}-\underset{s\;\in \;SLA}{\sum }\;PenaltyCost_{s}\\ & -CostOfTriggeredRetrievals\\ & -CostOfAutomaticRetrievals \end{array}$$

where $RequestIncome_{s}$ is the income received by CoaaS for all requests for each *s* ∈ SLA, computed as follows:

(8) $$RequestIncome_{s}=RequestNumber_{s}\times RequestPrice_{s}$$

Moreover, $PenaltyCost_{s}$ is the penalty cost incurred for SLA_s_ misses, computed as follows:

(9) $$PenaltyCost=MR_{s}\times RequestNumber_{s}\times PenaltyCost_{s}$$

CostOfAutomaticRetrievals (Equation (7)) is the cost incurred by the total number of retrievals occurred according to the end of the refresh period.

The main challenge in the application of the formula in (Equation (7)) is the dependence of the $PenaltyCost_{s}$ as well as the CostOfTriggeredRetrievals on the miss rate. The number of automatic retrievals also depends on the number of cache misses and shifts that are caused by these misses.

There are two more parameters defined for each SLA: the freshness period and the expected number of requests. The freshness period T defines for how long, since the retrieval of a data item, the item can be reused. The expected number of requests represents the number of requests for a particular SLA, which we expect to arrive during a planning period based on the statistical data from the performance datastore—the timescale database used to store the performance metrics of all the operations in CoaaS.

In the next section, we research the problem of finding the needed components for the application of the generalised formula in real scenarios. We start with the simplest scenario—a single SLA policy and then proceed to a more complex scenario, where two SLAs are defined.

### 4.1. A Policy with One SLA

**Figure 9:**
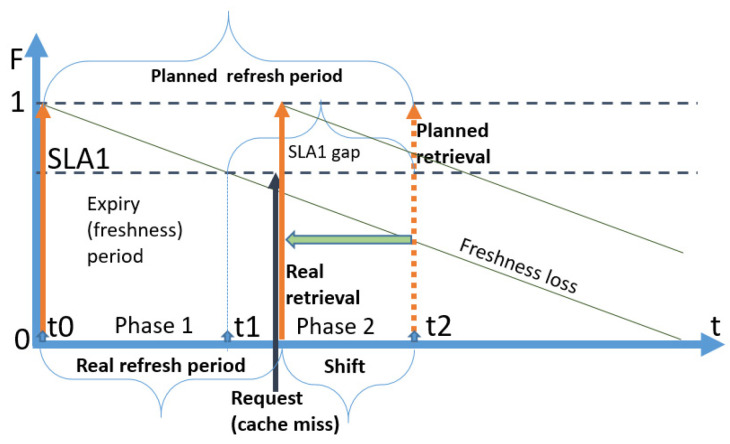
Cache miss caused a shift in the retrieval moment.

A detailed diagram of a planned refresh period for a policy with one SLA (1SLA) with a cache miss and a shift is presented in Figure 9. The process starts at time t = 0 with a retrieval, which is depicted by an orange arrow. The freshness of the cached attribute equals 1.0 at this stage. The next retrieval is planned at time t2. The end of the freshness period happens at time t1, and the time between t1 and t2 is the size of a gap. When a request arrival (depicted by a black arrow) happens between t1 and t2 (black arrow), an unplanned (i.e., triggered) retrieval is triggered.

The time between the moment of the unplanned retrieval and the time of planned retrieval (depicted by the horizontal green arrow) is the shift. Note that now there is no real need for a refresh t2, as the value will still be fresh enough by that time.

**Figure 10:**
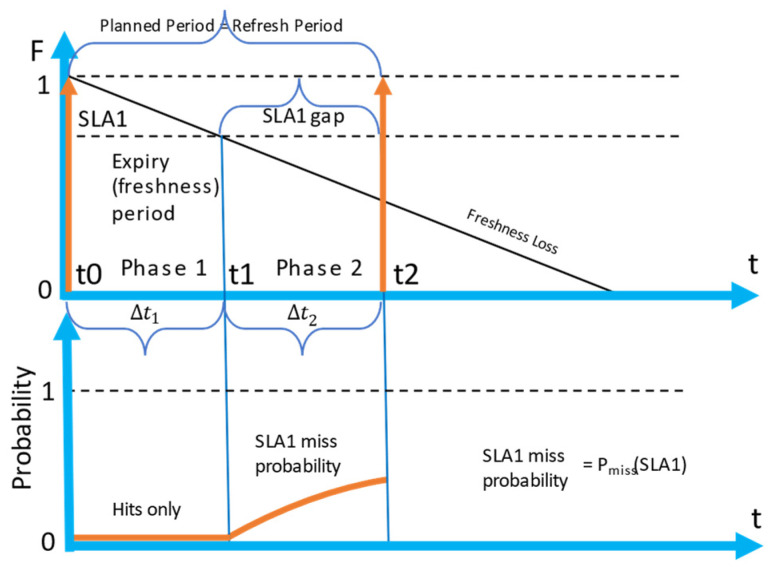
Segments (phases) of one refresh period and the cumulative probability distribution of a context cache miss.

In Figure 10, the detailed diagram of one full refresh period (top graph), and the corresponding cumulative distribution of miss probability during one refresh period (bottom graph) is presented.

During the first segment (referred to as Phase 1 in the figure), when the freshness of the attribute value is above the SLA threshold, only cache hits are possible. After t1, segment 2 (Phase 2) starts. In segment 2, the probability of meeting the first request arrival, which will cause a cache miss, grows exponentially according to the feature of the Poisson process:

(10) $$P_{miss}\left(t_{miss}\geq t_{1}\right)=1-e^{-\lambda t_{1}}$$

In the expression above, *t* is the time that has passed after $t_{1}$, and $t_{miss}$ is the time of arrival of a request causing a miss. When the second retrieval happens due to the moment of planned retrieval or a miss, the process will start from the beginning.

We should note that $P_{miss}$ represents the cumulative probability. It represents the chance of encountering the arrival before the time t, but not exactly at time t. The possible range of $P_{miss}$ is depicted in Figure 10 as $P_{miss}\left(SLA1\right)$. In the case, when a planned retrieval is set too far (reactive strategy), the cumulative probability of a cache miss will reach 1.

Our main interest is to estimate the cost of operation for a planning period. The general expression for estimating the cost is presented below:

(11) $$\begin{array}{ll} ProfitOfPlanningPeriod & \\ & =\overset{n}{\underset{1}{\sum }}RequestPrice\\ & -\overset{k}{\underset{1}{\sum }}PenaltiesCost\\ & -\overset{l}{\underset{1}{\sum }}CostOfTriggeredRetrievals-\overset{j}{\underset{1}{\sum }}CostOfAutoRetrievals \end{array}$$

In the expression above, *n* is the number of requests, *k* is the number of misses, l is the number of retrievals that are caused by a cache miss and *j* is the number of retrievals that happen according to the planned time. Here, we separate the cost of triggered retrievals from the cost of auto retrievals for two main reasons: first, clarity of explanation and, secondly, the potential use for more complex scenarios, where automatic retrievals are performed in advance, and therefore, can be served cheaper, as they may potentially use another interface for retrieval or different processing facilities.

In summary, we can say that the total cost of operation has one positive and two negative components. The positive component is the sum of prices, which are paid by consumers requesting context attributes. The negative components are (i) the sum of penalties, caused by cache misses, and (ii) the sum of retrieval costs.

The cost of serving requests in the case of full coverage (no gap) or the case of the reactive strategy (infinite gap) was already described in Section 3.1 and Section 3.2 correspondingly. However, in the case when there is a finite gap (*proactive strategy*), the computation becomes not as obvious. The main issue is that every time a miss happens, the whole picture shifts to the left along the time axis. Eventually, it is not clear how many refresh periods will fit in a planned period. Consequently, we can conclude that if we can find the hit rate, the miss rate, and the real number of refreshes, we will be able to estimate the cost.

Next, we can transform a formula for the cost of the planning period (Equation (11)) into a more usable form. The concept is based on finding the **H**it rate (HR), the **M**iss rate (MR), and the ratio of **R**efreshes to requests (RR). We called the formula of our approach the **HMR formula**. Details of the formula and its components are described below:

(12) $$\begin{array}{lll} ProfitOfPlanningPeriod & \\ & =HR\left(T,t_{g}\right)\times RequestNumber\times RequestPrice & \\ & +MR\left(T,t_{g}\right)\times RequestNumber\times RequestPrice & \\ & -MR\left(T,t_{g}\right)\times RequestNumber\times Penalty & \\ & -RR\left(T,t_{g}\right)\times RequestNumber\times RetrievalPrice \end{array}$$

The *hit rate*, *miss rate*, and *refresh ratio* are dependent on the length of the freshness period (*T*), and the size of a chosen gap, also referred to as gap time ($t_{g}$). As $HR\left(t_{g}\right)+MR\left(t_{g}\right)=1$, we can simplify the expression above by replacing the first two components with $\left(RequestNumber\times RequestPrice\right)$, consequently:

(13) $$\begin{array}{lll} ProfitOfPlanningPeriod & \\ & =RequestNumber\times RequestPrice & \\ & -\;\mathrm{MR}\left(T,t_{g}\right)\times RequestNumber\times Penalty & \\ & -RR\left(T,t_{g}\right)\times RequestNumber\times RetrievalPrice \end{array}$$

The positive component of the expression in Equation (13) represents the revenue, which CMPs receive from serving a certain number of requests (RequestNumber) for a specific price (RequestPrice), which arrive during the planning period. The two negative components are the penalties and the price of data retrieval. The penalties can be expressed as the number of all queries, multiplied by the miss rate, and multiplied by the cost of each penalty. It means that to find the penalty component, we need to find the miss rate.

While the first two components in Equation (13) are intuitively clear, the retrievals component is harder to define. The problem is that retrievals can happen because of (i) cache misses, (triggered retrievals), or (ii) as the result of reaching the end of the gap, (planned retrievals). For the cost estimation, it does not matter what type of retrieval happens. We only need the number of all retrievals that are expected to occur during the planning period. We have found that a convenient way to find the number of all retrievals is to express them through a ratio coefficient, which represents the ratio of retrievals to incoming requests. We call this coefficient the *Refresh ratio* and designate it as RR. It is vital to note that, while the hit and miss rate can only take values between 0 and 1, the refresh ratio can take any positive real value (${\mathbb{R}}^{+}$). Eventually, the third component of Equation (13) can be calculated as the refresh ratio multiplied by the number of requests and the price of one data retrieval.

We have described the meaning and the general components of the HRM formula; now, we can proceed to find the HR, MR, and RR, which are needed to apply the HRM formula.

To find the hit rate (HR(T, $t_{g}$)) for a policy with gaps we can use the expressions for HR(T) and MR(T), which we used for the reactive strategy (Equations (4) and (5)) together with the feature of the Poisson process, which describes the probability of the first arrival after a random point in time ($P=1-e^{-\lambda t}$). Hence, the expected hit rate and miss rate can be calculated as follows:

(14) $$HR\left(T,\;t_{g}\right)=\frac{E\left[N\left(T\right)\right]}{E\left[N\left(T\right)\right]+1-e^{-\lambda \;t_{g}}}$$

(15) $$MR\left(T,\;t_{g}\right)=\frac{1-e^{-\lambda \;t_{g}}}{E\left[N\left(T\right)\right]+1-e^{-\lambda \;t_{g}}}$$

In the expression above, $N\left(T\right)$ represents the number of requests, which will arrive during the freshness period of a cached data item.

An expected number of requests per real refresh period is defined as:

(16) $$E\left[ReqPerRealRefreshPeriod\right]=E\left[N\left(T\right)\right]+1-e^{-\lambda \;t_{g}}$$

In the formula above, by real refresh period, we mean the average time between retrievals, either triggered or automatic.

In this case, the refresh ratio (RR) is defined as:

(17) $$RR\left(T,\;t_{g}\right)=\frac{1}{E\left[N\left(T\right)\right]+1-e^{-\lambda \;t_{g}}}$$

Multiplying RR(T, $t_{g}$) by the expected number of requests during the planning period gives us the number of retrievals that happen during this period:

(18) $$RetrievalNum=RR\left(T,\;t_{g}\right)\times RequestNum$$

Now we have obtained all the required parts for the HMR formula, and it is possible to compute the overall cost of serving requests to a data item for a planned period for any given gap size *t_g_*.

**Figure 11:**
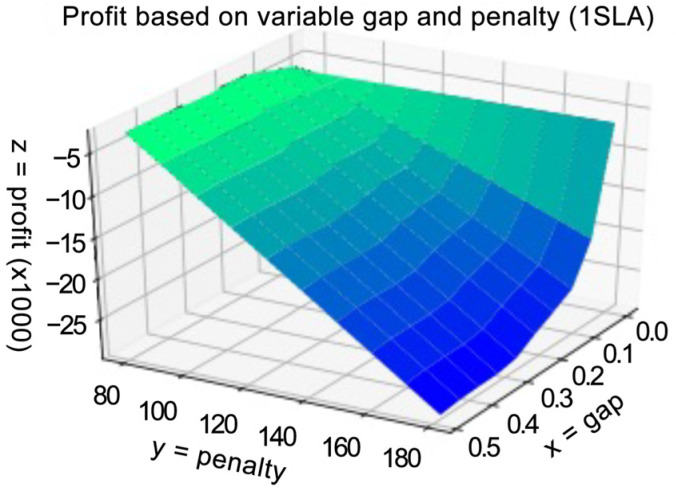
Profit for various gaps and their respective penalties.

We have run a set of simulations to check the components and the whole HMR formula. The results of this simulation show that the results obtained analytically closely match the results of our experiment. In Figure 11 the dependency of profit, gap size, and penalty are illustrated.

In the next section, we are progressing from a simple 1SLA policy to more advanced and realistic scenarios with multiple SLAs.

### 4.2. A Policy for Two SLAs (2SLA)

**Figure 12:**
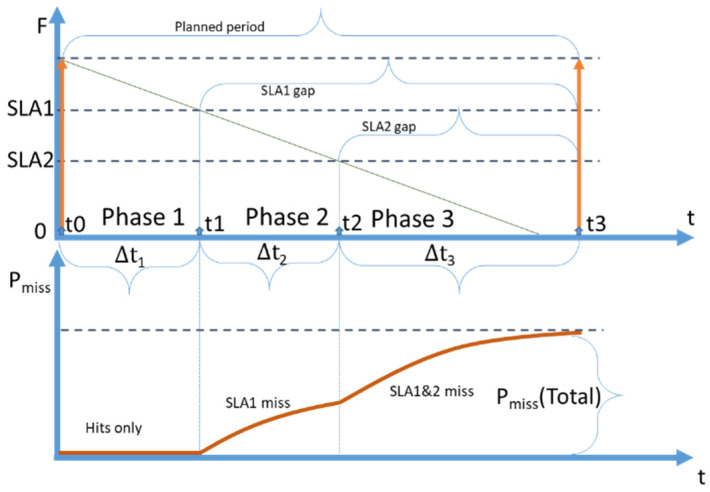
Segments (Phases) of one refresh period with 2SLA policy and probability of a miss.

In this section, we describe a scenario with two SLAs as an example of a situations when multiple SLAs are defined. In Figure 12, a detailed diagram of one refresh period (top graph) and the corresponding cumulative probability of a miss (bottom graph) for a scenario with two SLAs are presented.

The main difference between the 2 SLA policy (Figure 12), compared to the 1SLA policy (Figure 10), is that there is no single expiry period. SLA1 and SLA2 expiry periods are overlapping; however, the SLA2 expiry period is longer. That leads to a situation when an SLA1 request arriving during the second part of the SLA2 freshness period causes a cache miss. In the diagram presented in Figure 12, we have identified three segments (phases) of the process.

Segment 1 is the state when all the SLAs are covered by the cache, and a miss cannot happen. Segment 2 is the state when SLA1 is not covered (SLA1 gap), but the data are still fresh enough for SLA2. In segment 3, both SLAs are not covered (SLA1 and SLA2 gap) and any arrival of a request would cause a cache miss.

The bottom graph shown in Figure 12 represents how the cumulative probability of obtaining a cache miss is changing over time. During segment 1, the probability equals zero, then, during segment 2, it grows exponentially, as SLA1 requests can cause cache misses. In segment 3, the cumulative probability is also growing exponentially, but faster, and both SLA1 and SLA2 requests can cause a cache miss.

We can rewrite the formula of the cost for the planned period in the following way:

(19) $$\begin{array}{l} ProfitOfPlannedPeriod=\sum_{1}^{n}RequestPrice_{SLA1}+\sum_{1}^{m}RequestPrice_{SLA2}-\\ \sum_{1}^{k}PenaltiesCost_{SLA1}-\sum_{1}^{r}PenaltiesCost_{SLA2}-\\ \sum_{1}^{l}CostOfTriggeredRetrievals-\sum_{1}^{j}CostOfAutoRetrievals \end{array}$$

In the expression above, *n* is the number of SLA1 requests, *m* is the number of SLA2 requests, *k* is the number of SLA1 misses, *r* is the number of SLA2 misses, *l* is the number of retrievals that are caused by a cache miss, and *j* is the number of retrievals that occur according to the planned time.

**Figure 13:**
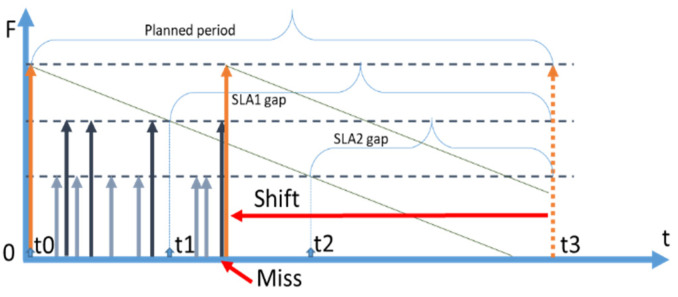
SLA1 request causes a miss during the second segment (phase).

**Figure 14:**
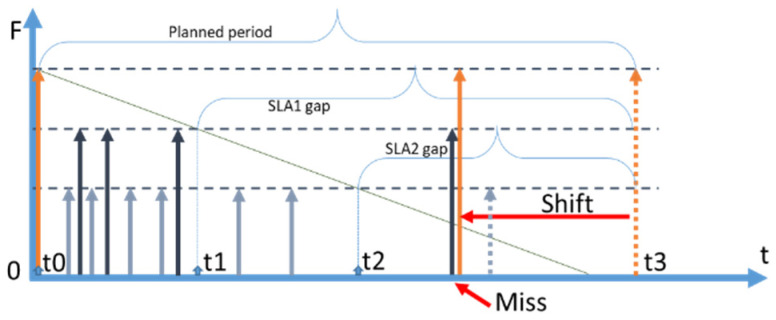
SLA1 request causes a miss during the third segment (phase).

Misses can be caused by SLA1 and SLA2. SLA1 request arrival can cause a miss in segment 2 and segment 3. Illustrations of possible options where SLA1 request causes a miss in 2 SLA scenario are presented in Figure 13 and Figure 14.

In Figure 13, a situation where an SLA1 request is arriving during segment 2 and causing a cache miss is shown.

**Figure 15:**
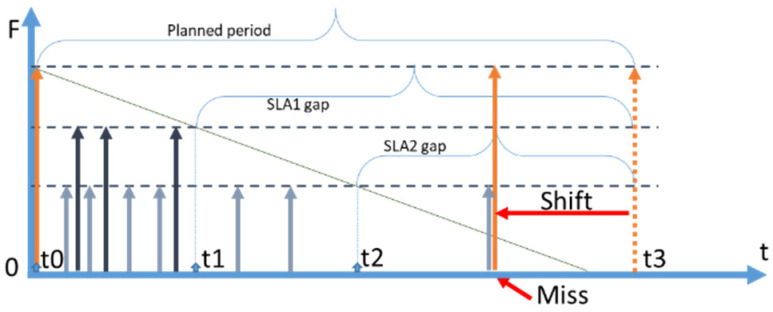
SLA2 request causes a miss during the third segment (phase).

In Figure 14, an SLA1 request arrives during the third segment, which is also causing a cache miss. An SLA2 request can cause a miss only during the third segment, as it is represented in Figure 15.

As it is shown in Figure 12, each refresh period consists of three segments. In segment 1, all the incoming requests are served from the cache without any misses. In segment two, all SLA2 requests are served from the cache. However, any SLA1 request is causing a cache miss and retrieval with a corresponding shift. In segment 3, any incoming request causes a cache miss and retrieval.

Next, we consider what happens if we put the moment of an automatic retrieval inside each of the segments:

1. *Retrieve in* segment *1*: Retrieving a data item before the end of segment 1 is not efficient, as the increase in the cost of retrieval is not compensated by an increase in income. Consequently, it makes sense to put the retrieval time only at the end of the expiry period of SLA1. It will provide full coverage for all SLAs.
2. *Retrieve in* segment *2:* The moment of planned retrieval lies between the end of the expiry period of SLA1 and the end of the expiry period of SLA2, all the SLA2 requests are served from the cache, but any SLA1 request causes a miss and requires a retrieval. It means that we can look at this scenario in the same way as we did in a scenario for 1SLA. The only difference with the 1SLA scenario, is that the total revenue will increase by the amount of average SLA2 queries served between the SLA1 expiry period and the first SLA1 request.
3. *Retrieve in* segment *3:* The moment of planned retrieval is set after the end of both SLA1 and SLA2 expiry periods. It means that during segment 1 all the requests are served from the cache; during segment 2, only SLA2 requests are served from the cache, and during segment 3 any incoming request will cause a cache miss and a retrieval.

In the next section, we develop a method for finding the profit of a planning period when *n* SLA is defined.

### 4.3. A Policy with Multiple SLAs or n SLAs (nSLA)

Let us consider one hypothetical refresh period (RP) and its realisation of profit. Let *ProfitRP* denote the realised value of profit in RP and $N_{s}$ denote the number of requests within RP relating to SLAs. Then, the expected value of *ProfitRP* can be expressed as follows:

(20) $$E\left[ProfitRP\right]=\sum_{s}\left(E\left[N_{s}\right]\times RequestPrice_{s}-E\left[M_{s}\right]\times Penalty_{s}\right)-CostOfRetrieval$$

where $M_{s}$ is the realised number of misses in RP, and CostOfRetrieval is the cost of retrieval happening at the beginning of the period.

**Figure 16:**
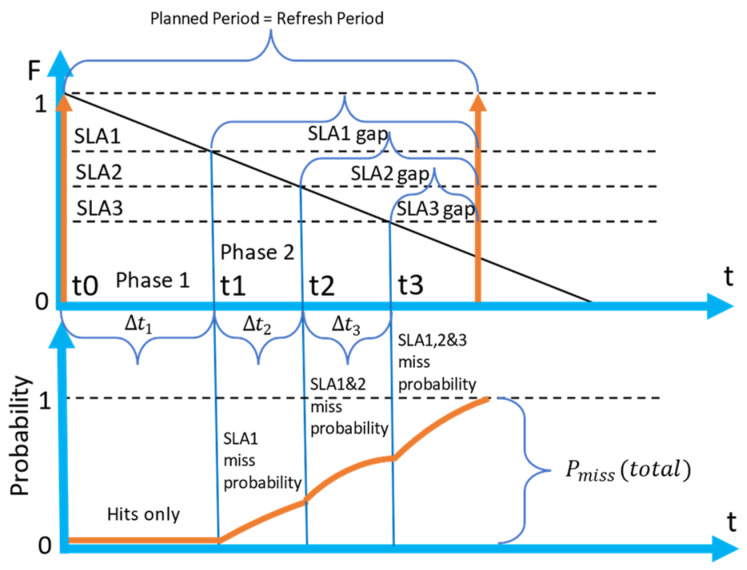
Segments (Phases) of one refresh period with nSLA policy and probability of a miss using a 3SLA scenario as an example.

Let $K\in \left\{1,\ldots ,\;n+1\right\}$ denote the number of segments in $\left[0,\;t_{max}\right]$ with distinct sets of fresh SLAs, where $t_{max}$ is the moment of planned retrieval. That is, segment *k* = 1 has all SLAs fresh, *k* = 2 has all but SLA1 fresh, *k* = 3 has all but SLA1 and SLA2 fresh etc. Note that *K* can be smaller than *n* if $t_{max}\leq \;t_{n-1}$. For example, consider Figure 16 where an automatic refresh happens between points *t_1_* and *t_2_*.

Then, $E\left[M_{s}\right]=\underset{k}{\sum }E\left[M_{sk}\right]$, where *M_sk_* is the realised number of misses of SLA *s* in segment *k*. Let *Miss_k_* be the hypothetical event that a miss happens in segment $k\;\in \left\{1,\ldots ,\;K\right\}$, which assumes there was no miss in segments {1,…,*K* − 1}. Obviously, $P\left[Miss_{1}\right]\equiv 0$. Moreover, let $Miss_{sk}$ be the event that the miss in segment *k* is triggered by a request of SLA *s*. Then, the expected number of misses of SLA *s* in segment *k,*

(21) $$E\left[M_{sk}\right]=\left\{\begin{array}{c} 0,when\;k\leq s,\\ P\left[Miss_{k}\right]\times P[Miss_{sk\;}|\;Miss_{k}],\;\mathrm{otherwise}. \end{array}\right.$$

Note that, due to the property of independent interfering Poisson processes (assuming requests for different SLAs are independent),

(22) $$P[Miss_{sk\;}|\;Miss_{k}]=\frac{\lambda_{s}}{\lambda_{1}+\cdots +\lambda_{k-1}}$$

where $\lambda_{i}$ represents the arrival rate of requests relating to SLA *i.*

Let *Miss_m..l_*(*u*, *v*) be the event that one of the SLAs in the range from *m* to *l* causes a (first and only) miss in the interval [*u*, *v*], assuming that $u\geq \;t_{l}$ (i.e., the freshness of these SLAs expired before *u*). The conditional probability of *Miss_m..l_(u, v),* under the condition of no misses before *u*, is as follows (using Equation (10)):

(23) $$P\left[Miss_{m..l}\left(u,v\right)\right]=1-e^{-\sum_{k=m}^{l}\lambda_{k}\left(v-u\right)}$$

Let $NoMiss_{1..k}$ be the event that no miss happens in any of the segments from 1 to *k*. Then the probabilities of no misses happening for each segment till segment *k* is a result of a sequential set of independent events. So, the $P\left[NoMiss_{1..k}\right]$ can be obtained by applying the product rule of probability of no misses in each segment. Therefore,

(24) $$P\left[NoMiss_{1..k}\right]=\overset{k}{\underset{q=1}{\prod }}\left(1-P\left[Miss_{1..q-1}\left(t_{q-1},t_{q}\right)\right]\right)$$

The probabilities of events $Miss_{m..l}\left(u,v\right)$ and $NoMiss_{1..k}$ enable the computation of the unconditional probability of $Miss_{k}$ as:

(25) $$P\left[Miss_{k}\right]=NoMiss_{1..k-1}\times P\left[Miss_{1..k-1}\left(t_{k-1},\;t_{k}\right)\right]$$

i.e., the unconditional probability of a miss in segment $k\;\in \left\{1,\ldots K\right\}$ is the product of conditional probabilities of no misses in the preceding segments 1...*k* − 1, times the conditional probability of a miss in segment *k*. Equations (22)–(25) enable the computation of *E*[*M_sk_*] (Equation (21)).

Similarly, $E\left[N_{s}\right]=\sum_{k}E\left[N_{sk}\right]$, where *N_sk_* is the realised number of requests of SLA *s* in segment *k*. Obviously, $E\left[N_{s1}\right]=\lambda_{s}t_{1}$ in the very first segment (as we used in Equations (14) and (15)).

Let $p_{1..l}^{Miss}\left(t\right)$, for $t\geq t_{l}$, be the probability density function of a miss at time *t* caused by a request of any SLA in the range from 1 to *l*. Clearly,

(26) $$p_{1..l}^{Miss}\left(t\right)=P\left[NoMiss_{1..l}\right]\times \left(\overset{l}{\underset{k=m}{\sum }}\lambda_{k}\right)\times e^{-\sum_{k=m}^{l}\lambda_{k}\left(t-t_{l}\right)}$$

i.e., it equals the probability of no misses before $t_{l}$ multiplied by the probability density function in the interval [$t_{l},\infty )$, shifted to [0,∞).

Moreover, let $N_{s}\left(u,v\right)$ be the realised number of requests of SLA *s* in the time [*u*,*v*], with $E\left[N_{s}\left(u,v\right)\right]=\lambda_{s}\left(v-u\right)$. Then,

(27) $$E\left[N_{sk}\right]=\left\{\begin{array}{c} E\left[N_{s}\left(0,t_{1}\right)\right]=\lambda_{s}t_{1},\;\;\;\;\;k=1;\\ \int_{t_{k-1}}^{t_{k}}p_{1..k-1}^{Miss}\left(t\right)\times E\left[N_{s}\left(t_{k-1},t\right)\right]dt+\\ P\left[NoMiss_{1..k}\right]\times E\left[N_{s}\left(t_{k-1},t_{k}\right)\right],\;\;\;\;2\leq k\leq s;\\ E\left[M_{sk}\right],\;\;\;\;k>s,\; \end{array}\right.$$

where the case 2≤k≤s was computed by the law of total expectation.

Note that an automatic refresh time can be chosen before the end of the expiry period of one or several SLAs. In such a case, we reset the expiry period of affected SLAs to the time of the planned auto refresh.

Then, using Equations (21), (26) and (27), we can compute $E\left[N_{s}\right]$ using:

(28) $$\begin{array}{ll} \int_{t_{k-1}}^{t_{k}}p_{1..k-1}^{Miss}\left(t\right)\times & E\left[N_{s}\left(t_{k-1},t\right)\right]dt\\ & =\overset{k-1}{\underset{q=1}{\prod }}\left(1-P\left[Miss_{1..q-1}\left(t_{q-1},t_{q}\right)\right]\right)\\ & \times \int_{t_{k-1}}^{t_{k}}\left(\overset{k-1}{\underset{q=1}{\sum }}\lambda_{q}\right)\times e^{-\sum_{q=1}^{k-1}\lambda_{q}\left(t-t_{k-1}\right)}\lambda_{s}\left(t-t_{k-1}\right)dt \end{array}$$

The integral in the right-hand side of Equation (28), substituting $\bar{\lambda }=\sum_{q=1}^{k-1}\lambda_{q}$ and $\bar{t}=t-t_{k-1}$, equals,

$$\begin{array}{ll} \int_{0}^{t_{k}-t_{k-1}}\bar{\lambda }e^{-\bar{\lambda \;}\bar{t}}\lambda_{s}\bar{t}d\bar{t} & =-\lambda_{s}\left(\bar{t}e^{-\bar{\lambda \;}\bar{t}}+\frac{1}{\bar{\lambda }}e^{-\bar{\lambda \;}\bar{t}}\right){|}_{0}^{t_{k}-t_{k-1}}\\ & =-\frac{1}{\bar{\lambda }}\lambda_{s}\left(\bar{\lambda }\left(t_{k}-t_{k-1}\right)e^{-\bar{\lambda \;}\left(t_{k}-t_{k-1}\right)}+e^{-\bar{\lambda \;}\left(t_{k}-t_{k-1}\right)}-1\right)\\ & =\;\frac{\lambda_{s}}{\bar{\lambda \;}}\left(1-\left(\bar{\lambda \;}\left(t_{k}-t_{k-1}\right)+1\right)\times e^{-\bar{\lambda \;}\left(t_{k}-t_{k-1}\right)}\right) \end{array}$$

We have computed the expected profit per refresh period. To compute the profit for a fixed time interval (whole planning period of a context data), note that the expected duration of a refresh period can be found as:

(29) $$E\left[RP\right]=E\left[\frac{N_{s}}{\lambda_{s}}\right],\;s\in \left\{1,\ldots n\right\}$$

## 5. Evaluation

We used the system design of Context-as-a-Service (CoaaS) [11], which was designed and developed by our research group to test our methodology. The main motivation behind developing CoaaS is to provide a generic and standard way to define, advertise, discover/acquire, and query context information. In other words, CoaaS facilitates context exchange between IoT entities.

### 5.1. Experimental Setup

Currently, the standardisation efforts in the area of CMPs and context query languages are led by the ETSI CIM working group [28], where the NGSI-LD language originating from the FIWARE Orion [12] platform is the basis for the proposed standard. In CoaaS, the module that is responsible for data storage, caching, and retrieval is called the Context Storage Management System (CSMS) [29]. The query interface is based on a specifically designed Context Definition and Query Language (CDQL) [11]. Situations are inferred based on the Context Spaces Theory [30]. The main service, which is potentially commercialisable, is the access to contextual information, which is retrieved and processed by the platform. At the same time, this context is created from data which are supplied by external providers. In this sense, CoaaS provides an advanced API to IoT data.

**Figure 17:**
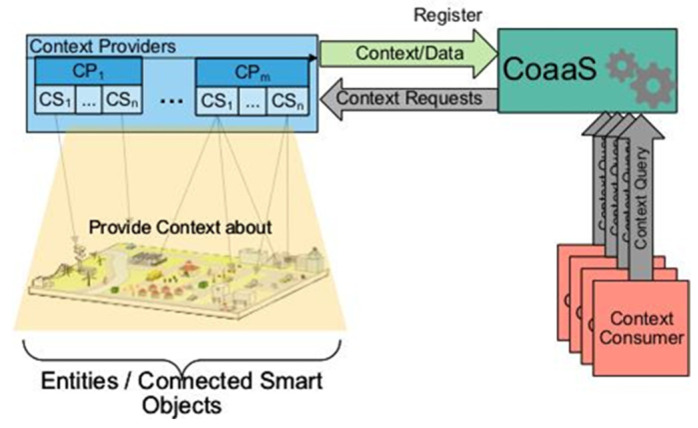
An overview of Context-as-a-Service in the IoT ecosystem.

Figure 17 depicts an overview of the CoaaS platform in an IoT ecosystem. As it is shown, context consumers send their contextual requirements to CoaaS as context queries, which are represented in CDQL. CDQL supports two types of queries, PULL-based query, where the query is only executed once on-demand, and PUSH-based query which enables continuous situation monitoring and asynchronous context delivery.

When the CoaaS platform is deployed for operation in an IoT ecosystem, none of the context consumers or context providers have full control over the platform. The platform provides access to contextual information by returning results of consumers’ queries; thus, the platform provides a service, that can be billed accordingly (e.g., per query or consumed resources). We used the context query load from [11] to test our proposed technique. The same use cases,

- Use case 1: School safety (context-aware school pickup management)—a smart IoT system that determines the safe alternatives to pick up children from school when the parent, guardian, or a pick-up person is late to arrive;
- Use case 2: Smart car parking recommendation—a context-aware IoT application that makes personalized suggestions of available parking slots to drivers or autonomous self-driving cars based on user preferences, car specifications, and related environmental conditions such as weather; and
- Use case 3: Smart car pre-conditioning—smart connected electrical vehicle pre-heating or cooling appropriately before a journey based on context related to the car’s location, driver’s location, driver’s calendar, weather conditions, etc.

were considered. For further details about these use cases, query execution in CoaaS, and the definition of CDQL language, please refer to our previously published papers [11]. In this paper we focus on modelling the cost-efficiency of data retrieval and caching in CoaaS; nevertheless, the presented methods can be used in other CMPs as well because we implement a generic context retrieval strategy in our proof-of-concept. Context providers were setup and simulated using the IoT data simulator (https://github.com/IBA-Group-IT/IoT-data-simulator, accessed on 6 June 2023). Behaviours of the context providers were inspired by the real-world testing performed in [11].

To simulate the query load, which would be coming from a query engine to Context Storage and Management System (CSMS) in CoaaS in real life, we need to generate a stream or a dataset of CDQL requests, that are aiming to access a context attribute with a certain level of freshness. We intentionally separated experimental measurements from the real CoaaS platform prototype, to avoid potential influence caused by the interference of unforeseen factors. However, as we presented in our previous works [15,22,25], our proposed refreshing strategies and configuration can be used in real CMPs such as the CoaaS where previous versions have been tested with large real-world motivated scenarios, e.g., context-aware smart car parking recommendation [15].

The distribution of request inter-arrival times should follow the Poisson law. We realised the dataset generator in Python 3, the generator can be accessed at GitHub (https://github.com/coaasCache/nSLA_method, accessed on 1 September 2023). As an input for the generation, it is required to provide the number of SLAs and the length of the simulated period in seconds. The prices of requests, penalties, and freshness period can be randomly generated or specified by a user. As a result of the generation, we obtain a set of events (arrival times) for each SLA. The generation is followed by a function, which analyses the profit of a planning period for a chosen gap size. We have also realised the method, presented in the previous section as an algorithm in Python 3. Both methods allow us to predict the profit; however, the simulation requires more computational resources and performs much slower, especially for cases, when the request arrival rate or the number of SLAs is high. The matching results obtained from both methods prove the correctness of our method. We have also created a notebook for comparing the results obtained from a simulation and analytical solution graphically. Below, we present some of the results, which illustrate the variety of possible outcomes, based on randomly selected SLAs.

### 5.2. Evaluation of the Computation Method for nSLA Policy

In this section, we evaluate the method for estimation of the cost of a planning period in the case of an nSLA policy according to the methodology which was proposed in Section 4.3. The solid red lines in the figures below represent the calculated metrics using our proposed theory (Equations (20)–(29)).

**Figure 18:**
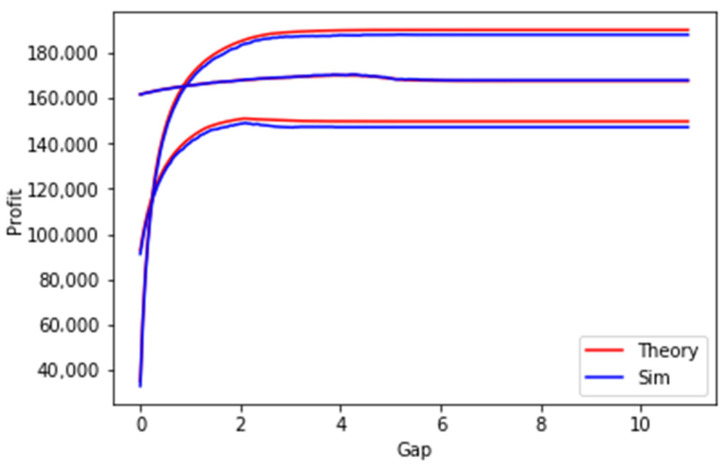
Comparison of theoretical and simulated monetary profits made by the CMP using the proactive context cache refreshing with shift.

In Figure 18, the results of three experiments are shown. In these experiments, we randomly generated freshness periods, request prices, and penalties for 10 SLAs as can be seen in the two experiments the maximum profit is achieved in the NOD-NOR mode (gap > 0 and gap < infinity). It can also be seen that the results of the simulation and the computation based on the developed method are closely matching as it also could be seen in Table 1. RMSE is the root-mean-square-error and RMSPE is the root-mean-square-percentage-error.

**Figure 19:**
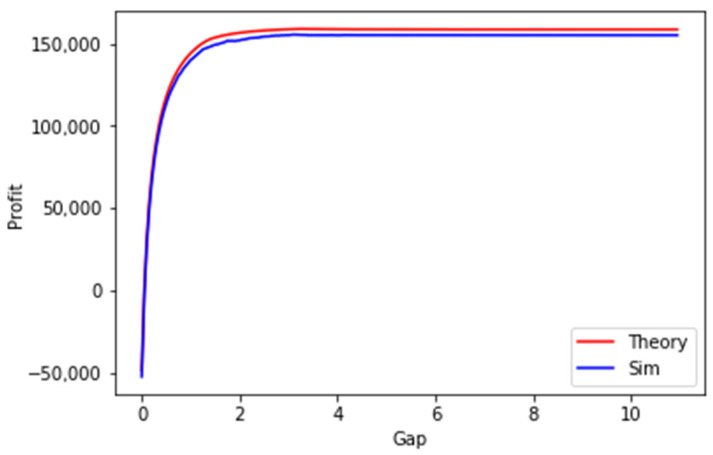
Theoretical and simulated results from an experiment allowing negative profit.

In Figure 19, the results of an experiment are shown, where the randomly generated SLAs allow potential negative profits, in case when the database strategy (gap = 0) is chosen. The difference in profits in this case is very significant. RMSE between the results is 3676.77±22.47, RMSPE is 0.02475, and the t-value is $1.52\times 10^{-230}$.

**Figure 20:**
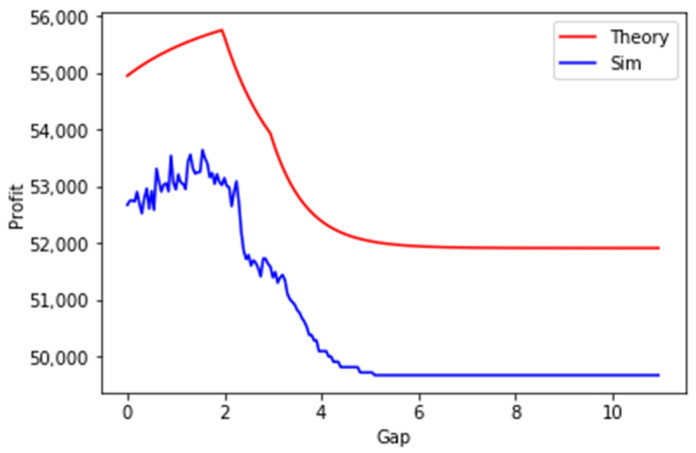
Sharp maximum in an experiment with 3 SLAs.

In Figure 20, the results of an experiment for 3 generated SLAs are presented. As can be seen, there is a sharp maximum of profit for a gap of two seconds. Both graphs have the same shape; however, there is some discrepancy between the theoretical and simulated results. The inaccuracy of simulation results is caused by a low number of events on a short planning period, resulting in a higher number of cache misses and triggered retrievals resulting in a higher probability of delay to respond with context (as against the relevant SLAs)—see Equation (27). As we show in the next section, allowing more time to optimize the retrieval period during a longer planning period, allows to alleviate this discrepancy. RMSE between the results is 2297.51±10.76, RMSPE is 0.0454, and the t-value is $7.74\times 10^{-256}$.

#### Testing for Longer Planning Periods

**Figure 21:**
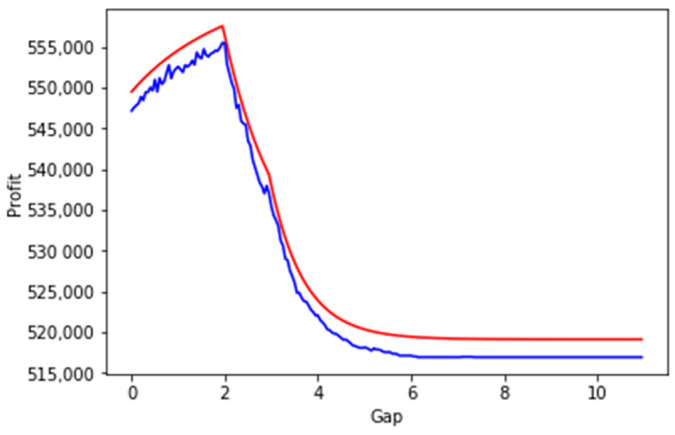
Experiment with 3 SLAs and a long planning period.

In the next experiment, we increased the length of the planning period to 6000 s from the original 600 s. Other parameters stayed unchanged. As can be seen in Figure 21, the results of the simulation and theory-based results match closely. RMSE between the results is 2292.59±22.63, RMSPE is 0.0044, and the t-value is $6.28\times 10^{-185}$.

In this section, we have evaluated the refresh rate-based caching strategies by comparing the analytical methods, which we proposed in Section 4 with the results of the simulation. We also provided a graphical comparison of the results for various gap sizes and other input parameters, to prove the applicability of the proposed methods with a variety of parameters. We have shown that there exist input parameters with which every strategy (full coverage, proactive, and reactive) is beneficial, and it is essential to have a methodology for optimal decision making.

We found that the proposed methods are producing reliable results which closely match the results of the simulation. It is proved in the above results where all t-value →0. It indicates that there is no significant difference between the estimated profits using our model and the simulated results using CoaaS, hence verifying our model.

## 6. Related Work

In this section, we briefly analyse the existing works related to caching and prefetching data in various middleware systems.

One of the main differences between a CMP with many other types of data-centric systems is the lack of a clearly defined pattern in data retrieval and ingestion [2]. A typical database is usually designed and maintained to serve a particular application or a limited set of applications. As a result, such databases can be tuned for specific loads. However, a modern CMP should automatically adapt to the load, which is generated by context consumers (queries) and context providers (ingestion). We cannot rely on manual tuning of a CMP for a particular use case or a set of use cases because (i) a CMP should show adequate performance in a wide range of use cases that are useful to the consumers, and (ii) CMPs are required to adapt fast and flexibly for variations in situations that manual tuning cannot facilitate.

The freshness of context (which inversely correlates with the age of context), its accuracy, completeness, and the latency of access to context, along with several other parameters, form the metric called Quality of Service (QoS).

Linking these groups of parameters is achieved by establishing Service Level Agreements (SLAs) between the context consumers, the middleware, and context providers. Then, these parameters can be used to build cost-based models used for cache management, prefetching, and other self-adaptation tasks of the CMP.

The area of cache management for IoT data, where the freshness, cost, latencies, undefined patterns of consumer requests, and other specific parameters important for CMP operation are considered is not well researched [6]. The problem becomes even more complicated when multiple SLAs are taken into account. For instance, consumers can subscribe to a platinum, golden, or silver plan, or even define their own specification of acceptable data freshness and associated costs. We investigated this problem of data retrieval, context caching cost analysis, and modelling in this paper.

Given the lack of previous work in context caching, we investigated the related work in data caching. There, we can distinguish this related work designed for fixed-size systems between *basic caching policies* and *intelligent policies.*

*Basic policies.* The most common examples of traditional cache replacement policies are LRU, LFU, SIZE, GD-size, GDSF, and their variations [31,32]. The main factors, which influence the decision about the eviction of an object from the cache are: (i) the *recency* of access to an object, (ii) the *frequency* of access to an object, (iii) the *size* (in bytes) of an object, (iv) the *cost* of retrieving the object, (v) the *latency* of access to an object when retrieved from an external source. The LRU (Least Recently Used) algorithm chooses objects for eviction which were requested least recently [33]. There exists a number of extensions for the LRU algorithm, e.g., LRU-threshold, SB-LRU, HLRU, LRU-hot, TRLU, and Pitkow/Recker.

LFU (Least Frequently Used) is a frequency-based algorithm which evicts the objects that were accessed less often. However, LFU suffers from the cache pollution problem; the objects, which were accessed many times long ago, are kept in the cache and occupy the space. The variations in LFU are such policies as LFU-aging, Window-LFU, HYPER-G, and LFU-DA.

The policy which has the largest size to free the storage resources called SIZE evicts the objects. SIZE suffers from cache pollution and poor performance because of not considering the latency and cost of re-obtaining the large objects. The extension of the SIZE policy is the Greedy-Dual-Size (GDS) algorithm [34]. There also exists a group of policies called Randomised policies where the choice of an object for eviction is randomised. Examples of such policies are RAND, LRU-C, and HARMONIC. These policies can provide a simple way to clear the space in the cache for new items; however, the performance of such policies is questionable. It is also hard to figure out clearly the advantages and disadvantages of these policies. While the above-described group of algorithms can bring benefits in certain scenarios, they cannot guarantee any kind of optimality. In that sense, we can call them heuristic-based methods. These types of algorithms have no self-adaptation and the designer, or the administrator of a system must evaluate and compare the performance of these algorithms in case he/she wants to adopt these policies in the system under consideration.

*Intelligent policies.* The emergence of machine learning (ML) technologies led to the appearance of intelligent caching policies [7], which use access logs as training data. For instance, artificial neural networks (ANN) were used for making cache decisions in proxy caches [35,36]. Another approach based on the logistic regression technique (LR) was proposed in [37]. A similar aim was pursued in [38], but the back-propagation neural network was used instead of the LR.

There also exist works showing the applicability of genetic algorithms to cache replacement [39]. The main criticism of the usage of ML-based approaches to large caches is that the learning process can take a significant amount of time and computational resources [40]. Moreover, quick adaptation to changes in load is also challenging for such methods.

According to [31,41,42] there exist several main measures (metrics) that can be used to analyse the performance of a chosen caching strategy in a certain environment. These metrics are the Hit Rate (HR) and its inverse, the Miss Rate (MR). These metrics are often also referred to as the Hit Ratio and Miss Ratio. Other important metrics are the Byte Hit Rate (BHR) and the Latency Saving Rate (LSR).

*Prefetching.* Data caching is typically a reactive technique, and the decisions are made on which objects that have already been obtained by the server should be kept and reused, and which objects should be evicted. When the system predicts future requests, the needed data can be retrieved from the sources and preprocessed and an incoming query can be served with less latency. Mostly, the prefetching approaches are based on analysing the content of objects or the history of access to objects. In [43], a double dependency graph (DDG) was used to manage the prefetching decisions. Another popular approach to predicting access to objects is the Markov Model (MM)-based approach. For instance, such an approach was used in [44,45]. Another group of prefetching algorithms is based on the computation of the cost function. There exist works where the prefetching decision is based on the popularity of objects [44], the lifetime of objects [25], or a balance of popularity and update rate [46]. There also exists a group of algorithms called Objective-Greedy prefetching [47], which aims to improve a chosen metric by prefetching such objects that will maximise the chosen metric. There are also works employing data mining [48] and clustering-based prefetching [49] to improve the performance. One of these works, for instance, employs the page-rank algorithm for clustering web objects [50].

Surveys of performance efficiency criteria for web prefetching are available in [31,43]. The main metrics are (i) precision, (ii) byte precision, (iii) recall, (iv) byte recall, (v) traffic increase, and (vi) latency ratio.

*Caching of IoT data*. The IoT data differ from many other data types, as they are transient. Moreover, the consumer can also be interested in receiving data with a certain level of precision. There exists a number of works on caching IoT data in Information Centric Networks (ICN) or Named Data Networks (NDN). Originally, NDN was designed to support fast access to immutable objects (e.g., video streaming). Meddeb et al. [24,51] showed that the caching nodes of ICN can also be used to store IoT data. The classification of IoT traffic was proposed by Liu [52]. Meddeb et al. proposed the Least Fresh First (LFF) caching strategy for IoT data in ICN. The LFF strategy is based on evicting those values from the caching node, which have the lowest freshness. They propose an algorithm to predict T_fresh_, the period when the data item is considered fresh. The algorithm is based on the Autoregressive Moving Average (ARMA) model [53].

Al-Turjman et al. [23] proposed the Least Value First (LVF) policy for ICN caching nodes. It is a function-based caching approach that takes into account the delay of data fetching, popularity, and age parameters for deciding the cache eviction. The proposed utility function assigns a value to each object. Al-Turjman et al. in general defined a Delay Model, a Popularity Model, and an Age model. A formula for computing the value of a data item is presented below (Equation (26)).

(30) $$Value_{NDO_{\;i}}=\alpha \times D_{i}^{\prime }+\beta \times Pop_{NDO_{\;i}}+\gamma \times Drop_{NDO_{\;i}}$$

In the formula above, *D* represents the delay model, which is calculated based on the latency of access to the data source providing this data item; *Pop* represents the frequency of queries to the data item; *Drop* represents the age of the data item, which is proportional to the TTL. Parameters α, β, and γ are introduced for manual tuning purposes. The authors showed the superiority of the LFF strategy compared to LFU and LRU. However, as the costs are not taken into account in this model, it is not possible to apply it for multiple SLAs, and its application to non-fixed size systems is also questionable.

*Caching Strategies for Elastically Scalable Cloud-based Systems.* The paradigm shift for cache management when moving from fixed-size systems to cloud systems was highlighted in [54,55,56]. Cloud systems usually rely on the pay-per-use model. Eventually, the cache management problem is not limited by the size of storage or processing resources anymore. It is limited by monetary costs that allow to use cloud resources. Consequently, minimising the cost of using the cloud-based system while keeping a defined level of QoS becomes the main objective in the cloud paradigm. 

Le Scouarnec et al. [57] developed a cloud-oriented caching policy for video streaming services. In their model, the frequency of access to a cached data item and the cost of cloud services were the determining factors for cache management decisions. While the proposed models in [57] are valuable for video streaming services, they are not directly suitable for IoT data; as such, an essential parameter such as freshness is not taken into account. The latencies of access and their influence on the final cost are not taken into account as well.

*Probabilistic Approaches to Caching and Prefetching.* In [27], Jung et al. investigated the following problem: *how to predict the hit rate of a single data item, if the statistics of requests to this item and its TTL are known?*

The initial models used by Jung et al. were developed by Cohen et al. [58,59]. They have developed a simple model to predict cache hits in distributed caches for several specific different distributions of inter-request time.

Jung et al. [27] focused on a single cache but provided a model for predicting the hit rate for any arbitrary inter-request time distribution. The important assumption is that a sequence of requests to a particular data item can be represented as a sequence of random variables, which are independently and identically distributed (i.i.d.). This means that the process can be viewed as a renewal process [60]. Despite the approach being conservative and simple, Jung et al. reported good prediction accuracy.

The main finding of Jung et al. in [27] was the formulas for finding the hit and miss rates based on the expiry period (T) and the expectation of requests arriving during this period E[N(T)]. The formula for the hit rate is presented below [27]:

(31) $$\mathrm{HR}\left(T\right)=\frac{E\left[N\left(T\right)\right]}{E\left[N\left(T\right)\right]+1}$$

Based on the fact that MR(T) = 1 − HR(T), the miss rate can be found as follows [27]:

(32) $$\mathrm{MR}\left(T\right)=\frac{1}{E\left[N\left(T\right)\right]+1}$$

Scwefel et al. [61] and Olsen et al. [62] analysed the caching strategies and ways to establish the adaptive strategy for context middleware. They have looked at the problem from a different angle. In the proposed scenario, the mismatch probability between the value ‘contained’ in the source system, and the value ‘contained’ in the cache of the middleware was in the scope. In general, the described scenario is a reactive Quality of Service (QoS) -based strategy.

The authors defined three main performance metrics, which are (i) the *mismatch probability* (mmPr), (ii) the *access delay*, and (iii) the *network overhead.*

## 7. Conclusions and Further Work

In this paper, we focused on the refresh rate-based caching strategies and models, where the cache decision is expressed as the amount of time until the next retrieval, after the end of the freshness period for the most expensive SLA. We introduced three main cache refreshing strategies for a single context attribute, which are (i) full coverage, (ii) reactive, and (iii) proactive strategies. We have shown how to estimate the profit for the first and the second strategies based on the existing body of knowledge. The proactive strategy required developing a model to estimate the monetary profit from responding to context queries, which was presented in Section 4. This model is the main contribution of this paper. While requiring a more sophisticated methodology, when realised, the proactive strategy can help to reach the maximum profit of the CSMS operation. The evaluation of the proposed method is presented in Section 5.

In our future work, we will be developing methods for estimating the freshness of data items. We will also consider different distributions of arrivals, especially taking into account patterns of queries (e.g., data item d.i_.1_ is often queried after d.i._2_), or when several data items can only be retrieved together from the provider for a joint price.

Business requirements can change fast and there is a need to adapt the cache strategies accordingly. This can potentially be achieved with the application of modern machine-learning techniques. At the same time, maintaining the understanding of processes through obtaining an analytical solution, instead of purely relying on ML decisions, is also worth the effort, in our opinion.

The problem of defining the price both from the side of the providers of context and from the side of a CMP might also be an issue. For that, solving the inverse problem might be required (i.e., with fixed profit find suitable SLA parameters), as well as the development of visualising instruments and dashboards for the administrators and management of the IoT infrastructure.

## Figures and Tables

**Table 1:** Difference of model estimated profit against the simulated results.

Experiment	RMSE	RMSPE	t-Value
Exp 1	2452.70±18.56	0.0169	$3.17\times 10^{-210}$
Exp 2	2084.50±7.27	0.0115	$5.23\times 10^{-284}$
Exp 3	270.31±7.87	0.0016	$3.23\times 10^{-47}$

## Data Availability

Software, data, and results presented in this paper can be found at https://github.com/coaasCache/nSLA_method.
